# A Case–Control Study by ddPCR of ALU 260/111 and LINE-1 266/97 Copy Number Ratio in Circulating Cell-Free DNA in Plasma Revealed LINE-1 266/97 as a Potential Biomarker for Early Breast Cancer Detection

**DOI:** 10.3390/ijms24108520

**Published:** 2023-05-10

**Authors:** Marina Bortul, Fabiola Giudici, Domenico Tierno, Daniele Generali, Serena Scomersi, Gabriele Grassi, Cristina Bottin, Maria Rosa Cappelletti, Fabrizio Zanconati, Bruna Scaggiante

**Affiliations:** 1 Department of Medical, Surgical and Health Sciences, University of Trieste, 34149 Trieste, Italy; m.bortul@fmc.units.it (M.B.); dgenerali@units.it (D.G.); cbottin@units.it (C.B.); fabrizio.zanconati@asugi.sanita.fg.it (F.Z.); 2 Breast Unit, Azienda Sanitaria Universitaria Integrata Giuliano Isontina (ASUGI), 34149 Trieste, Italy; serena.scomersi@asugi.sanita.fvg.it; 3 Cancer Epidemiologic Unit, Centro di Riferimento Oncologico di Aviano (CRO), Istituto di Ricovero e Cura a Carattere Scientifico (IRCCS), 33081 Aviano, Italy; fabiola.giudici@cro.it; 4 Department of Life Sciences, University of Trieste, 34127 Trieste, Italy; domenico.tierno@phd.units.it (D.T.); ggrassi@units.it (G.G.); 5 Breast Cancer Unit and Translational Research Unit, Azienda Socio-Sanitaria Territoriale di Cremona-ASST, 26100 Cremona, Italy; mariarosacappelletti@gmail.com

**Keywords:** ALU, breast cancer, cfDI, cfDNA, copy number ratio, *EEF1A2*, LINE-1, ddPCR, liquid biopsy

## Abstract

Background: In Western countries, breast cancer (BC) is the most common cancer in women. Early detection has a positive impact on survival, quality of life, and public health costs. Mammography screening programs have increased early detection rates, but new approaches to more personalized surveillance could further improve diagnosis. Circulating cell-free DNA (cfDNA) in blood could provide a potential tool for early diagnosis by analyzing cfDNA quantity, circulating tumor DNA mutations, or cfDNA integrity (cfDI). Methods: Plasma was obtained from the blood of 106 breast cancer patients (cases) and 103 healthy women (controls). Digital droplet PCR was used for the determination of ALU 260/111 bp and LINE-1 266/97 bp copy number ratio and cfDI. cfDNA abundance was calculated using copies of the *EEF1A2* gene. The accuracy of biomarker discrimination was analyzed with receiver operating characteristic curve (ROC). Sensitivity analyses were performed to account for age as a potential confounder. Results: Cases had significantly lower ALU 260/111 or LINE-1 266/97 copy number ratios (median; ALU 260/111 = 0.08, LINE-1 266/97 = 0.20), compared with control (median; ALU 260/111 = 0.10, LINE-1 266/97 = 0.28) (*p* < 0.001). ROC analysis showed that copy number ratio discriminated cases from controls (area under the curve, AUC = 0.69, 95% CI: 0.62–0.76 for ALU and 0.80, 95% CI: 0.73–0.86 for LINE-1). ROC from cfDI confirmed the better diagnostic performance of LINE-1 compared with ALU. Conclusions: Analysis of LINE-1 266/97 copy number ratio or cfDI by ddPCR appears to be a useful noninvasive test that could aid in early BC detection. Further studies in a large cohort are needed to validate the biomarker.

## 1. Introduction

Breast cancer (BC) is the most common cancer diagnosed in women and its incidence continues to increase worldwide, leading to morbidity, disability, and mortality [1]. Early detection is crucial for the best prognosis and possible cure. In addition, early detection of BC has a positive impact on survival, patient quality of life, and public health costs.

Although mammography screening has significantly increased the detection rate of early-stage BC, many women already receive a diagnosis of a locally advanced or metastatic stage of the disease [2]. Some of these women do not participate in active surveillance due to their younger age, which has a detrimental effect on early detection [3].

New BC screening approaches for more personalized surveillance can be derived from circulating cell-free DNA (cfDNA) analysis. cfDNA has been used as a tool for diagnosis, prognosis, therapy selection, and relapse detection in many cancers, including BC [4,5,6]. The evaluation of cell-free DNA integrity (cfDI) is of particular interest as a biomarker because it is more sensitive than cfDNA quantity and potentially more representative of cancer heterogeneity than a single mutation [6].

The early detection of BC by cfDI and its value as a biomarker is not fully understood [6]. In particular, is not clear if cfDI increases or decreases in BC patients. We developed a digital droplet PCR analysis to investigate in the plasma of 106 patients and 103 healthy controls the cfDNA copy number ratio of ALU 260/97 and LINE-1 266/97 of longer over shorter targeted regions previously investigated by Madhavan et al. [7]. *Arthrobacter luteus* (ALU) sequences are short interspersed nuclear elements with a size of 300 bp that represent about 10% of the human genome. Long interspersed nuclear element 1 (LINE-1) has a size of 6000 bp and constitutes about 17% of the human genome. Both LINE-1 and ALU are mobile elements that contribute to genome variability but also play important functional roles in gene expression and epigenetic regulation. These elements are involved in the development of cancer by promoting genomic instability [8].

The development of reproducible, standardized methods for the detection and quantification of cfDNA is important to improve the sensitivity, specificity, and informative value of these potential biomarkers. Our primary objective was to investigate the copy number ratio and the cfDI of ALU 260 bp over 111 bp and of LINE-1 266 bp over 97 bp in plasma by ddPCR in study groups of early-stage BC patients compared to healthy controls. The aim was to test whether these targets could be confirmed by this more sensitive, accurate, and reproducible amplification method as biomarkers that could be used in routine clinical practice for early cancer detection. Secondary issues were (1) the relationship between clinicopathological behavior and ALU and LINE-1 plasma biomarkers; and (2) quantification of the amount of cfDNA in early-stage BC patients compared with healthy controls with *EEF1A2* gene copies, a target that we verified to have good accuracy for haploid genomes screened using ddPCR.

## 2. Results

### 2.1. Characateristics of the Study Population

Healthy females (*n* = 103) were women undergoing routine gynecological examination at the Breast Cancer Unit and Translational Research Unit of the Hospital of Cremona. The mean age of control women was 52 ± 11 (standard deviation, SD) years. BC patients (*n* = 106) had a primary breast cancer diagnosis at the Breast Cancer Unit of the Hospital of Trieste. The mean age was 62 ± 13 (SD). Table 1 shows the American Joint Committee on Cancer (AJCC) stage and histopathology characteristics of patients whose sera were sampled preoperatively. The breast cancers diagnosed in the case cohort were 8 (7.6%) in situ, 76 (77.6%) luminal-like, 12 (12.2%) HER2+, and 10 (10.2%) triple negative. In total, 34.0% of tumors were poorly differentiated (G3) and 46 women (43.4%) had Ki67 index >=20% that is a marker for the aggressiveness of cancer, recommended by the International Ki67 in Breast Cancer Working Group [9]. Most of the breast cancers were treated with conservative surgery (62.3%), tumor dimensions were mainly less than 2 cm (66.0%), and 82 (77.4%) were negative to lymph node sentinel biopsy. Population characteristics are fully reported in Table 1.

Luminal-like BC: includes luminal A and B subtypes, is characterized by the expression of estrogen receptor (ER) and/or progesterone (PR) and with HER2–.

### 2.2. Evaluation of ALU 260/111 and LINE-1 266/97 Copy Number Ratio in Plasma of BC Patients and Healthy Controls

The copy number ratios of ALU 260 bp over 111 bp (ALU 260/111) and LINE-1 266 bp over 97 bp (LINE-1 266/97) were determined in the plasma of patients and healthy controls by digital droplet PCR amplifying larger and shorter fragments with primer set pairs of Madhavan et al. [7] as described in Materials and Methods. This is because ratio alteration between longer and shorter fragments for a given target has been demonstrated in many cancers, including BC [6].

**Figure 1:**
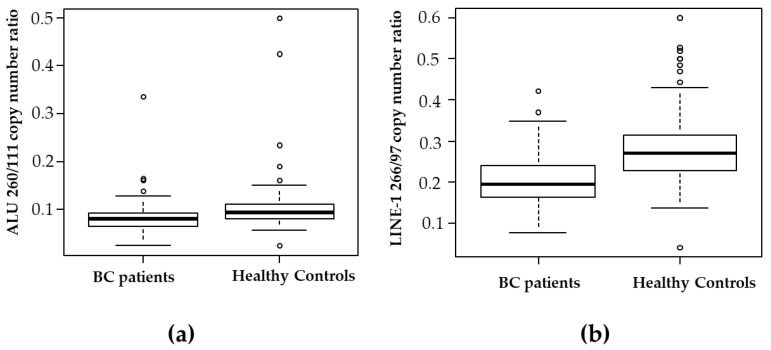
Box plot of copy number ratio of ALU 260/111 (**a**) and of LINE-1 266/97 (**b**) in plasma of Breast Cancer (BC) patients (*n* = 106) and healthy controls (*n* = 103) determined by ddPCR as described in Materials and Methods.

As shown in Figure 1a, ALU 260/111 copy number ratio was significantly lower in the case of BC patients group compared with the healthy control group (medians: 0.0795 vs. 0.0930, *p* < 0.001, Mann–Whitney test, see Supplementary Materials Table S1). To discriminate between BC patients and healthy controls, an optimal cutoff point was determined to be 0.0835, with a sensitivity of 61% (95% confidence interval (CI): 0.51–0.71) and a specificity of 72% (95% CI: 0.62–0.80).

The copy number ratio of LINE-1 266/97 is shown in Figure 1b. BC patients had lower values for LINE-1 266/97 than healthy controls (medians: 0.19 vs. 0.27, *p* < 0.001 Mann–Whitney test, see Supplementary Materials Table S2). To discriminate between the BC patients group and the healthy control group, an optimal cutoff point was determined to be 0.23 for LINE-1 266/97 with a sensitivity of 73% (95% CI: 0.64–0.82) and a specificity of 74% (95% CI: 0.64–0.83).

We found a correlation between ALU260/111 and LINE-1 266/97 copy number ratio in BC patients and healthy controls (rho = 0.37 and 0.50, respectively), both indicating the same effect to lower levels in BC patients.

**Figure 2:**
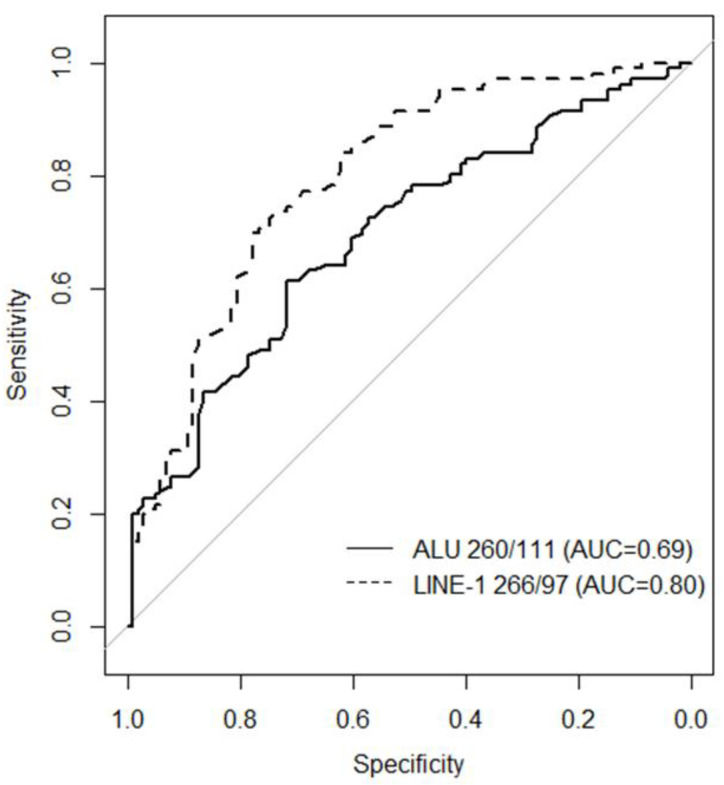
ROC analysis: Pairwise comparison of the ROC curves of ALU 260/111 and LINE-1 266/97 copy number ratio.

An analysis of receptor characteristics (ROC) was performed, taking into account the age difference between patients and controls. The ROC curves distinguishing between the BC patients and the healthy controls are shown in Figure 2. The AUC showed good accuracy for the ALU260/111 and LINE-1 266/97 copy number ratio to discriminate BC patients from healthy controls (see also Supplementary Materials Table S3). However, a comparison of the two ROC curves showed significantly better performance of the cfDI of LINE-1 266/97 (AUC = 0.80, 95% CI: 0.73–0.86) compared to ALU 260/111 (AUC = 0.69, 95% CI: 0.62–0.73; *p* = 0.0067, DeLong test; Supplementary Materials Table S1).

### 2.3. Correlation between Clinical Parameters and ALU 260/111 or LINE-1 266/97 Copy Number Ratio

In the BC patients group, the association between the ALU 260/111 or LINE-1 266/97 copy number ratio and other established clinical parameters, including tumor size, lymph node involvement, grading, and the status of estrogen receptor (ER), progesterone receptor (PR), and Her2/neu, is shown in Table 2.

ALU 260/111 copy number ratio values were significantly higher in women with positive lymph nodes (*p* = 0.002). The LINE-1 266/97 copy number ratio was lower in triple-negative breast cancer compared to the other molecular profiles, but without reaching statistical significance (*p* = 0.06). No other consistent associations with other factors were found.

### 2.4. Evaluation of the Cell-Free DNA Integrity (cfDI) of ALU 260/111 and LINE-1 266/97 in BC Patients and Healthy Controls

The cfDI was calculated for both ALU 260/111 and LINE-1 266/97 as described in Materials and Methods. To ensure the correctness of the calculation by ddPCR, we determined the cfDI on DNA from PBMCs derived from healthy donors. As shown in Table 3, both ALU and LINE-1 in PBMC DNA had a cfDI close to unity, as described by Madhavan et al. [7]. However, in the plasma of healthy controls, the cfDI was quite near the unit for LINE-1 but significantly lower for ALU as observed by other authors [7,10].

**Figure 3:**
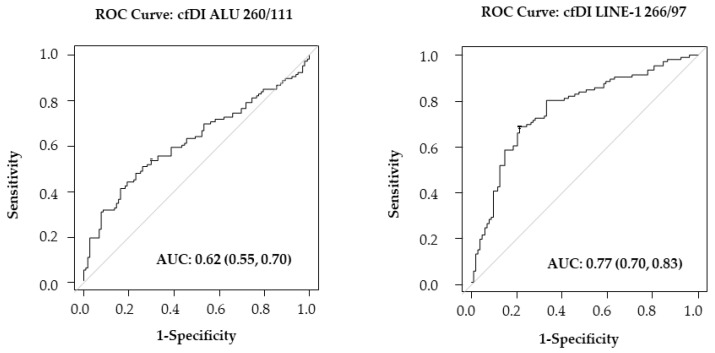
ROC analysis: ROC curves of cell-free DNA integrity (cfDI) of ALU 260/111 and LINE-1 266/97.

The ROC curves of cfDI calculated for ALU 260/111 and LINE-1 266/97 confirmed LINE-1 as a better biomarker to distinguish BC patients from healthy controls (Figure 3).

### 2.5. Deepening Larger or Shorter Fragments Quantity Variation in Copy Number Ratio

To understand whether the larger or shorter fragment amount resulted in copy number ratio variation between cases and controls, we assessed the amount of cfDNA in each sample by targeting *EEF1A2* gene copies by ddPCR assay for probe.

**Figure 4:**
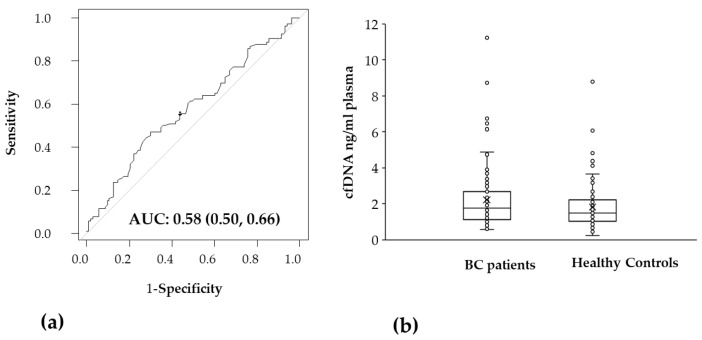
(**a**) ROC analysis of *EEF1A2* gene quantity determined by ddPCR assay for probe and expressed as ng/mL sample (**b**) Comparison of cfDNA quantity in plasma (ng/μL of plasma) of Breast Cancer patients and healthy controls determined from *EEF1A2* copies as described in Materials and Methods. BC, Breast Cancer; cfDNA, circulating cell-free DNA.

As shown in Figure 4a, we found that the *EEF1A2* amount (ng/μL of sample) did not significantly discriminate between cases and controls, although there was a slight increase in the amount in BC patients compared to healthy controls (median 0.0367125 vs. 0.0297000, *p* = 0.053). Total cfDNA concentrations, calculated from *EEF1A2* haploid genomes screened, were 2.2 ± 1.67 ng/mL plasma in BC patients and 1.82 ± 1.24 ng/mL plasma in healthy controls (Figure 4b) which is also consistent with other findings [5,11].

Therefore, we used the *EEF1A2* DNA quantity to normalize the copy number of ALU 260, ALU 111, LINE-1 266, and LINE-1 97 for each sample. As shown in Table 4, we found that the copy number of longer fragments of ALU and LINE-1 significantly decreased in BC patients compared with healthy controls (*p* < 0.0001).

## 3. Discussion

Long interspersed nuclear element 1 (LINE-1) repeats represent a family of active autonomous retrotransposons, also responsible for ALU retrotransposition, that are approximately 6000 bp long and account for about 17% of the human genome. In 50% of human cancers, an increase in LINE-1 transposable activity is observed, and their insertion in tumor suppression genes or their methylation status has been related to malignancy [8,12]. In this respect, many studies targeted ALU and LINE-1 repeats in cfDNA of BC patients to determine cfDI by quantitative PCR [7,13,14,15,16,17,18]. In BC patients, the majority of the studies targeted LINE-1 266 bp and 97 bp [7,14,16]; only Miao et al. targeted LINE-1 259 bp and 97 bp [17]. The targeting of ALU sequences in cfDNA of BC patients was ALU 245 bp and 115 bp [15,18] or ALU 260 bp and 111 bp [7,13,14,16]. In the plasma cfDNA of patients with early BC diagnosis or healthy control women study groups, we targeted ALU 260 bp and 111 bp and LINE-1 266 bp and 97 bp copy number by digital droplet PCR. To our knowledge, this is the first study using ddPCR to provide more sensitive and precise absolute quantification of target amplification technology. We found that BC patients have a significantly lower copy number ratio of ALU 260/111 and LINE-1 266/97 compared to healthy controls (see Figure 1). Analogously, the cfDI values of both ALU 260/111 and LINE-1 266/97 were lower in BC patients than in healthy controls in agreement with the findings of Madhavan et al. [7]. Of note, all studies with ALU 260/111 or LINE-1 266/97 observed a decrease in cfDI in cancer patients versus healthy controls [7,10,19], as we previously noted [6]. On the contrary, other targets such as ALU 245/115 [15,18], ALU 247/60 [20], and LINE-1 259/97 [17] showed an increase in the cfDI of cancer patients versus healthy controls. Thus, the difference seems to be related to the target, although the explanation deserves further investigation. In our study, the LINE-1 266/97 copy number ratio or the cfDI appear to be better predictors of early BC detection, discriminating cases from controls with an AUC of 0.80 and 0.77, respectively, versus ALU 260/111 with an AUC of 0.69 for copy number ratio and 0.62 for cfDI (see Figure 2 and Figure 3). The fact that we found a decrease in the cfDI accordingly to copy number ratio leads to the question of whether there was an increase in shorter fragments or longer ones. We found in both ALU and LINE-1 targets a significant decrease in longer fragments (see Table 4). Our results show that only the copy number of the longer fragments of both ALU and LINE-1 decreases in BC patients compared to healthy controls, resulting in a decrease in the ratio in BC patients. More evidence has suggested that cfDNA is more fragmented in cancer patients than in normal subjects [21,22]. Our findings agree with this observation and thus the increase in ctDNA fragmentation could decrease the copy number of longer ALU and LINE-1 amplicons. In our opinion, this phenomenon cannot be explained only by the contribution of cfDNAs smaller than 200 bp by the apoptotic death of cancer cells, as suggested by some authors [16,18,23,24,25]. We think that this could also be due to the higher and variable length fragmentation of cfDNA in cancer patients compared to healthy individuals, which has been observed by other authors who suggested mechanisms such as changes in chromatin structure (affecting nucleosomal organization), genetic and epigenetic aberrations, or different nuclease contents in cancer cells [22,26]. The reason why we did not find an increase in shorter fragments may be due to ctDNA fragmentation below 100 bp as demonstrated by Thierry et al. [21]. Currently, there is no further information on the mechanisms, but further studies on cfDNA liquid biopsy size fragments [27] may reveal the cfDNA fragmentomic signature in early BC cancer patients in the coming years.

The quantification of cfDNA by *EEF1A2* showed plasma quantities in healthy controls accordingly to our previous findings [5]. In healthy subjects, hematopoietic cells are the main source of the basal amount of cfDNA, but high variability of cfDNA quantity can be due to many causes including physical exercise or stress conditions [28] and this was also our evidence for some high cfDNA quantity values (see Figure 4). However, in BC patients the cfDNA quantity did not sufficiently discriminate between the two study groups (see Figure 4). Our data strongly agreed with the majority of patients having BC at early stages (in situ or <2 cm) supporting the idea that cfDNA quantity in advanced cancer is higher than in early ones [21].

The limitation of the present study is the small sample size of our study groups, which should be increased in future studies in order to validate LINE-1 266/97 as a biomarker for early cancer detection by ddPCR. However, the abundance of the LINE-1 target in cfDNA, as well as the possibility to analyze only larger fragments in a reproducible manner with ddPCR, opens new interesting perspectives in repeat sequence analysis in liquid biopsy.

## 4. Materials and Methods

### 4.1. Study Population

We performed a retrospective study analyzing blood samples collected before surgery from 106 women diagnosed with primary breast cancer at the Breast Cancer Unit of the Cattinara Hospital of Trieste. The study was approved by the Committee for Ethics of the Friuli Venezia Giulia country, Italy (ethical approval IRB: n. 2017-Os-102-ASUITS). The control group consisted of 103 healthy women undergoing routine gynecologic routine examination for prevention at the Breast Cancer Unit of the Hospital of Cremona, Italy (ethical approval protocol nr. Ex01/4111/04). Informed consent was collected from all study participants.

### 4.2. Plasma Preparation and DNA Extraction

Blood was obtained by venipuncture into a 10 mL Vacutainer K2-EDTA tube and processed within 1 h of collection. The blood was centrifuged at 3000× *g* for 10 min at 4 °C and the supernatant was then centrifuged at 12,000× *g* for 5 min at 4 °C. The plasma was aliquoted in cryovials and stored at −80 °C.

DNA extraction from the plasma sample (1.2 mL) was performed by automated MagCore ExtractorHF16 (Diatec Pharmacogenetics, Jesi, Italy) with MagCorePlasmaDNA Extraction kit 105 (Diatec Pharmacogenetics) following manufacturer instructions. The cfDNA was recovered in 60 μL of elution buffer. The sample was stored at −80 °C in a unique aliquot.

DNA from PBMCs from healthy donors was a gift from Prof. G. Grassi.

### 4.3. Digital Droplet PCR Quantification of ALU and LINE-1 Copy Number, cfDI, and EEF1A2 cfDNA Quantity in Plasma

The copy number of ALU 260 bp and ALU 111 bp and the LINE-1 266 bp and LINE-1 97 bp were determined by 2D analysis digital droplet PCR in Eva Green assay as indicated by Bio-Rad protocol. The primer sequences were the same as Madhavan et al. [7]. ALU260 and LINE-1 266 primers were at 250 nM in the reaction mixture; and ALU 111 and LINE-1 97 were 125 nM in the reaction mixture. The amplification conditions were 95 °C 5 min; 40 cycles: 95 °C 30 s, 56.5 °C 1 min; 4 °C 5 min; 90 °C 5 min. The best comparison between larger and shorter fragments was assured by dilution of the DNA sample to give a comparable number of positive droplets between larger and shorter fragments: 1:40 for ALU260, 1:360 for ALU 111, 1:5 for LINE-1 266, and 1:30 for LINE-1 97. To calculate the cfDNA quantity in a sample, we used the targeting of *EEF1A2* gene copies with a probe following Bio-Rad instructions. The amplification condition was 95 °C 10 min; 39 cycles: 94 °C 30 s, 57 °C 1 min; 98 °C 10 min. All samples had ≥9000 accepted droplets to be considered for analysis. The copy number of ALU 260 bp, ALU 111 bp, LINE-1 266 bp, and LINE-1 97 bp was determined by Quantasoft, the software coupled to the Droplet Reader QX200 that applies a Poisson algorithm to calculate the initial concentration of DNA target molecules as units of copies/μL input based on positive and negative droplets. Specifically, after setting an appropriate fluorescence threshold to determine the number of positive droplets (containing at least one target molecule), the software calculates the number of copies per μL of the reaction volume. As indicated by Tai et al. [29], this value is determined from the number of positive droplets and the total number of droplets (negative and positive) according to the following formula:

λ = −log(1 − p)

where λ is the average number of copies per droplet and p is the ratio of positive droplets to the total number, since the reaction volume per droplet, which is about 1 nl, is known (the software itself calculates the correct value).

In our case, the copies per μL of the sample were determined by dividing the value of copies in 20 μL (total reaction volume) determined by the software by the μL of the DNA sample added to the reaction mix. This value is then multiplied by the dilution coefficient, to obtain the number of copies per μL of the sample.

The *EEF1A2* copy number of each sample was used to normalize either ALU 260 and ALU 111 or LINE-1 266 and LINE-1 97 copy number among different samples to compare cases and controls.

The cfDI was calculated as follows:

cfDI ALU = copy number × 260/copy number × 111

cfDI LINE-1 = copy number × 266/copy number × 97

Amplicon lengths were included in the calculation of cfDI because ddPCR quantification provided us with the copy number value that must be converted to DNA quantity to compare our results with those obtained in the literature [7] based on amplicon concentration from qPCR. The cfDIs of ALU and LINE-1 were expressed as the ratio of concentrations (ng/μL). To obtain the concentration of each amplicon (ALU 111, ALU 260, LINE-1 266 and LINE-1 97), we used the following formula: ng/μL = copies/μL × bpn × 618 × 1.7 × 10^−15^, where bpn was the number of amplicon base pairs, 618 was the average weight of a base pair in daltons, and 1.7 × 10^−15^ was the conversion factor from dalton to ng of DNA. The last two factors were eliminated in the cfDI ratio since they were the same in the numerator and denominator of long fragments (ALU 260 and LINE-1 266) over short fragments (ALU 111 and LINE-1 97).

The cfDNA concentration in plasma was calculated by *EEF1A2* copy number considering that 1 haploid human genome is 3.3 pg of DNA and the QuantaSoft software readout (Quanta Soft 1.7.7.0917) of copies in 20 μL gives the number of haploid genomes screened, taking into account the concentration factor of the DNA extracted from the plasma.

### 4.4. Statistical Analyses and Data Visualization

We performed statistical analyses with R statistical software (R version 4.2.3, The R Foundation (https://www.R-project.org/)).

The clinicopathological characteristics were summarized using descriptive statistics: median and range for continuous variables after verifying the nonparametric distribution of data through Shapiro–Wilk test. Qualitative variables were expressed with absolute frequencies and percentages.

Median serum levels of cfDI of ALU and LINE-1 were analyzed compared to demographics, and tumor characteristics (type of surgery, tumor dimension, lymph nodal status, ki-67, and molecular profile) using Mann–Whitney tests for independent data for quantitative variables and chi-square tests or Fisher’s exact tests where appropriate for categorical parameters.

Median serum levels of cfDI of ALU and LINE-1 were compared between breast cancer women and healthy controls with Mann–Whitney tests. Receiver operating characteristic (ROC) analysis was carried out to assess the discriminatory power of cfDI concentration between cases and controls and the corresponding area under the curve (AUC) with a 95% confidence interval (95% CI) was calculated. The Sweets classification of accuracy was adopted to interpret the AUC values. An optimal cutoff able to maximize sensitivity (SE) and specificity (SP) was identified for each biomarker (R-package: “OptimalCutPoints”). ROC curves of the different biomarkers were compared to identify the best diagnostic test. The change in AUC was tested using the DeLong test [30]. Receiver operating characteristics (ROC) curve analysis was performed taking into account a difference in age between the patients and controls. The threshold for statistical significance was established at *p* < 0.05.

## 5. Conclusions

In the era of precision medicine that aims to improve cancer diagnosis and treatment through molecular information, investigations into the possible application of liquid biopsy as a noninvasive diagnostic tool are spreading. Liquid biopsy has the potential to address the identification of possible predictive biomarkers that may guide treatment decisions, monitoring of treatment response, and identification of resistance and disease recurrence. Among BC studies, fragmentomics with ctDNA and cfDI analysis have shown their role as valuable methods to extract information from the liquid biopsy with potential applications in the clinical setting from cancer detection to evaluation of treatment response and anticipation of recurrence diagnosis [31].

We developed a diagnostic tool based on digital droplet PCR quantification of ALU 260/111 and LINE-1 266/97 copy number ratio demonstrating, in a case–control population of BC patients at diagnosis, a good accuracy for both ALU260/111 and LINE-1 266/97 to discriminate BC patients from healthy controls, with better diagnostic performance of LINE-1 266/97 compared to ALU 260/111. This is the first study of ALU 260/111 and LINE-1 266/97 performed with ddPCR. This technique can guarantee absolute quantifications that are comparable among different laboratories and this is particularly relevant for the clinical application of liquid biopsy in screening or follow-up. Of note, the ddPCR led to highlighting that the longer fragments decrease in number in BC patients, thus lowering the copy number ratio. The rationale could be ascribed to the higher fragmentation of circulating cell-free DNA in cancer patients than in healthy controls [21,22].

Our work was performed by ddPCR for specific detection of LINE-1 and ALU in early-stage breast cancer patients, in whom we assume that the circulating tumor DNA quantity is very low. Compared with the study by Madhavan et al. [7], we found a higher discrimination value between cases and controls for LINE-1 compared with ALU targets, which may be related to the higher sensitivity of ddPCR compared with qPCR. In addition, the high precision and reproducibility of ddPCR were tested by repeating a series of different sample measurements at different time points, resulting in comparable results for either ALU and LINE-1 or *EEF1A2*. This may stimulate new interest in clinical validation of the methodology for liquid biopsy.

Larger studies are needed to validate LINE-1 266/97 as a predictive biomarker for BC onset in ddPCR and to contribute to filling the gap between research and clinical practice, with the perspective to offer patients new opportunities for early cancer detection and personalized diagnosis. However, in our opinion, this could be an interesting biomarker to further explore for application in screening programs for BC women.

## Figures and Tables

**Table 1:** Clinical and pathological characteristics of Breast Cancer (BC) patients. N, number of patients; SD, standard deviation.

Variable	Case Cohort (*n* = 106)
**Mean Age, years (SD)**	62 (13)
**Age (N,%)**	
<60	47 (44.3%)
≥60	59 (55.7%)
**Type of Tumor (N,%)**	
In situ	8 (7.6%)
Invasive	98 (92.4%)
**Surgery (N,%)**	
Conservative	66 (62.3%)
Mastectomy	40 (37.7%)
**Tumor dimension (N,%)**	
In situ	8 (7.6%)
<2 cm	70 (66.0%)
≥2 cm	28 (26.4%)
**Lymph node status (N,%)**	
N0	82 (77.4%)
N+	24 (22.6%)
**Ki-67 (N,%)**	
<20	60 (56.6%)
≥20	46 (43.4%)
**Grading (N,%)**	
G1	24 (22.6%)
G2	46 (43.4%)
G3	36 (34.0%)
**Molecular Profile Invasive BC (N,%)**	
Luminal-like	76 (77.6%)
Her2+	12 (12.2%)
Triple Negative	10 (10.2%)

**Table 2:** Correlation between clinical–pathological status and ALU 260/111 or LINE-1 266/97 copy number ratio.

Variable	ALU 260/111 Copy Number Ratio Median (Min-Max)	*p*-Value	LINE-1 266/97 Copy Number Ratio Median (Min-Max)	*p*-Value
**Age**				
<60	0.08 (0.04–0.16)	0.31	0.18 (0.10–0.35)	0.54
≥60	0.08 (0.03–0.34)		0.18 (0.08–0.42)	
**Type of Tumor**				
In situ	0.07 (0.03–0.11)	0.41	0.20 (0.08–0.26)	0.92
Invasive	0.08 (0.03–0.34)		0.19 (0.08–0.42)	
**Surgery**				
Conservative	0.07 (0.03–0.34)	0.53	0.20 (0.08–0.37)	0.43
Mastectomy	0.08 (0.04–0.16)		0.19 (0.08–0.42)	
**Tumor dimension**				
in situ	0.07 (0.03–0.11)	0.70	0.20 (0.08–0.26)	0.44
<2 cm	0.08 (0.04–0.34)		0.20 (0.08–0.35)	
≥2 cm	0.08 (0.03–0.16)		0.19 (0.08–0.42)	
**Lymph node status**				
N0	0.07 (0.03–0.34)	0.002	0.19 (0.08–0.37)	0.62
N+	0.09 (0.04–0.16)		0.20 (0.10–0.42)	
**Ki67**				
<20	0.07 (0.03–0.34)	0.53	0.20 (0.08–0.37)	0.64
≥20	0.08 (0.04–0.16)		0.19 (0.08–0.42)	
**Grading**				
G1–G2	0.08 (0.03–0.34)	0.06	0.20 (0.08–0.37)	0.94
G3	0.09 (0.04–0.16)		0.20 (0.08–0.42)	
**Molecular Profile**				
Luminal-like	0.08 (0.04–0.34)	0.73	0.20 (0.08–0.42)	0.06
Her2+	0.08 (0.04–0.11)		0.18 (0.10–0.30)	
Triple Negative	0.08 (0.04–0.12)		0.15 (0.10–0.25)	

**Table 3:** Mean cfDI ± standard deviation (min and max values in parenthesis) of ALU and LINE-1 from different study groups. cfDI was obtained from the concentration ratio (ng/µL sample) of ALU 266 on ALU 111 and of LINE-1 266 on LINE-1 97. * *p* < 0.002; ** *p* < 0.001 Mann–Whitney test. BC, Breast Cancer; cfDI, cell-free DNA integrity; PBMC, Peripheral Blood Mononuclear Cells.

Study Groups	cfDI ALU 260/111	*n*	cfDI LINE-1 266/97	*n*
*PBMC from healthy donors*	0.85 ± 0.12 (0.74–0.99)	5	1.14 ± 0.13 (1.04–1.29)	3
*Healthy Controls*	0.23 ± 0.09 (0.06–0.72)	103	0.77 ± 0.45 (0.15–3.97)	103
*BC Patients*	0.17 ± 0.25 (0.02–2.21) *	106	0.53 ± 0.25 (0.08–1.66) **	106

**Table 4:** Mean number of copies/µL sample of ALU 260, ALU 111, LINE-1 266, and LINE-1 97 from different study groups. Each value has been normalized on the corresponding number of copies of *EEF1A2*/ µL sample. The values are reported as median ± standard deviation (min and max in parenthesis). * *p* < 0.0001 Mann–Whitney test. BC, Breast Cancer; n, number of cases or controls.

Study Groups	ALU 260	ALU 111	LINE-1 266	LINE-1 97	*n*
*Healthy controls*	14,404 ± 5673 (3500–37,629)	148,630 ± 50,708 (25,791–306,666)	1744 ± 673 (330–4481)	6193 ± 2145 (1163–14,733)	103
*BC patients*	11,445 ± 7904 * (3200–82,377)	154,339 ± 112,700 (14,462 –715,800)	1207 ± 385 * (155–2325)	6295 ± 1843 (2601–11,955)	106

## Data Availability

Not avaible for privacy.

## Supplementary Materials

The supporting information can be downloaded at https://www.mdpi.com/article/10.3390/ijms24108520/s1.
