# Supplementary material for: A Case–Control Study by ddPCR of ALU 260/111 and LINE-1 266/97 Copy Number Ratio in Circulating Cell-Free DNA in Plasma Revealed LINE-1 266/97 as a Potential Biomarker for Early Breast Cancer Detection
Source: Int J Mol Sci. 2023 May 10;24(10):8520. doi: 10.3390/ijms24108520 (PMC10217920; doi:10.3390/ijms24108520)
Supplement: Supplementary file 1 [file ijms-24-08520-s001.zip › ijms-2378756-supplementary.pdf]

Table S1. ALU260/111 copy number ratio in BC patients and Healthy controls

	min	median	max
**BC patients**	0.03	0.0795	0.34*
**Healthy controls**	0.02	0.093	0.5

*p<0.001, Mann-Withney

Table S2 . LINE-1 266/97 copy number ratio in BC patients and Healthy controls

	min	median	max
**BC patients**	0.08	0.19	0.20*
**Healthy controls**	0.04	0.27	0.6

*p<0.001, Mann-Withney

Table S3. Pairwise comparison of ROC curves

Variable (copy number ratio)	AUC	SE	95% CI	***p*-value**
**ALU 260/111**	0.69	0.04	0.62 to 0.76	0.0067
**LINE-1 266/97**	0.80	0.03	0.73 to 0.86	

p=0.0067, De-Long
